# *Pneumocystis jirovecii* pneumonia in a patient with HIV infection: complex diagnosis using Giemsa-stained bronchoalveolar lavage fluid

**DOI:** 10.1590/0037-8682-0150-2021

**Published:** 2021-04-28

**Authors:** Louisy Sanches dos Santos, Lincoln de Oliveira Sant’Anna, Max Roberto Batista Araújo

**Affiliations:** 1 Universidade do Estado do Rio de Janeiro, Faculdade de Ciências Médicas, Departamento de Microbiologia, Imunologia e Parasitologia, Rio de Janeiro, RJ, Brasil.; 2 Instituto Hermes Pardini, Núcleo Técnico Operacional, Setor de Microbiologia, Vespasiano, MG, Brasil.

**FIGURE 1:**
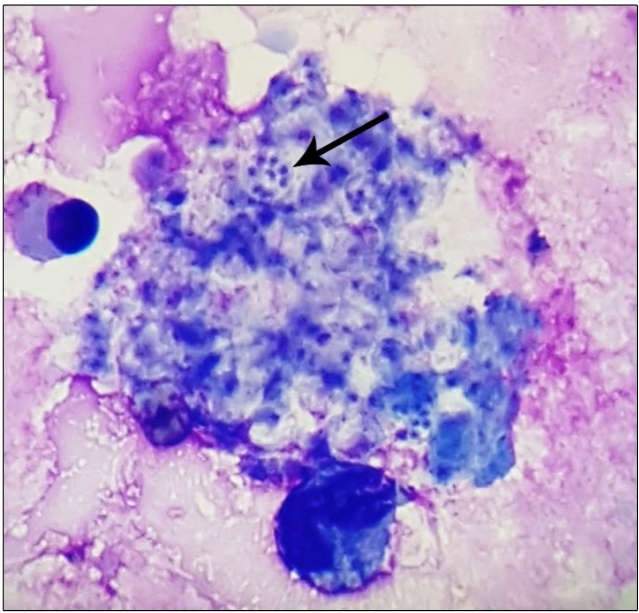
Giemsa staining (original magnification, ×1000) of bronchoalveolar fluid showing cyst forms of *Pneumocystis jirovecii* (black arrow).

A 54-year-old Brazilian man presented to the emergency department with cough, chest pain, high fever, and dyspnea. He had no history of sexually transmitted infections. A thoracic computed tomography scan showed ground-glass opacification areas and mediastinal lymphadenopathy. Laboratory tests revealed the following abnormalities: absolute monocyte count,80 cells/mm^3^; partial pressure of oxygen, 55.3 mmHg; and C-reactive protein,304.3 mg/L. Blood tests for cytomegalovirus*, Chlamydia pneumoniae*, *Legionella pneumophila*, and *Mycoplasma pneumoniae* and a sputum analysis for *Mycobacterium tuberculosis* were negative. Microscopic examination of Giemsa-stained bronchoalveolar lavage fluid (BALF) showed cysts of the atypical fungus *Pneumocystis jirovecii* (Figure 1), the etiological agent of pneumocystis pneumonia (PCP). Additional investigations revealed human immunodeficiency virus (HIV) infection, low CD4^+^T-cell count (128 cells/mm^3^), and increased lactate dehydrogenase levels. Antiretroviral therapy (ART) and trimethoprim/sulfamethoxazole (14 days) treatment were established. The patient was discharged 30 days post-admission.

PCP is a life-threatening infection that is often observed in immunocompromised individuals. Although the incidence has decreased among HIV-infected individuals due to the widespread use of ART and prophylaxis, PCP remains the most prevalent opportunistic infection among HIV-infected patients worldwide and persists as the main acquired immunodeficiency syndrome-defining infection^1^.

Due to non-specific signs and symptoms, and because *P. jirovecii* cannot be cultured in artificial media, the diagnosis of PCP is challenging. Methods involving DNA detection and serological biomarkers are available, but the microscopic observation of *P. jirovecii* in BALF is still the gold standard for PCP diagnosis^1^ ^,^ ^2^.
